# Society Meetings

**Published:** 1896-08

**Authors:** 


### Society Meetings.

### AMERICAN ASSOCIATION OF OBSTETRICIANS AND GYNECOLOGISTS.

The ninth annual meeting of the American Association of Obstetricians and Gynecologists will be held at the Hotel Jefferson, Richmond, Va., Tuesday, Wednesday and Thursday, September 22, 23 and 24, 1896.

The proprietors of the "Jefferson" offer special rates to the Fellows of the association, their families and guests, as well as to any physicians who come to attend the meeting. It is confidently expected that the railways will offer transportation at a uniform rate of a fare and a third on the certificate plan to all in attendance. Let all obtain certificates from their local ticket agents, or from the nearest point where certificates are granted.

### OUTLINE PROGRAM.

The association will meet in executive session with closed doors on Tuesday, September 22d, at 9.30 o'clock A. M., for the election of new Fellows. The open session for the reading of papers will begin at 10 o'clock A. M. Recess for luncheon at 1 o'clock P. M. Afternoon session at 3 o'clock P. M. An evening session will be held Tuesday at 8 o'clock.

Morning session will begin Wednesday at 9.30 o'clock for the reading of scientific papers. Recess at 1 o'clock. Afternoon session at 3 o'clock. Adjournment at 5 o'clock. Executive session at 6.30 o'clock. Annual dinner at 8 o'clock P. M.

Thursday morning the session will begin at 10 o'clock. Recess at 1 o'clock. Afternoon session at 3 o'clock. Final adjournment at 5 o'clock. A full attendance is specially requested at the final session.

### PAPERS PROMISED.

NOTE.—No attempt is made to arrange papers in the order in which they are to be read. That will be done in the permanent program.

1. Principles and progress in gynecology. President's address. Joseph Price, Philadelphia. 2. Vaginal hysterectomy by the clamp method, Sherwood Dunn, Los Angeles. 3. Further experience with appendicitis, A. Vander Veer, Albany. 4. Relation of malignant disease of the adnexa to primary invasion of the uterus, A. P. Clarke, Cambridge. 5. Treatment of puerperal septicemia, H. W. Longyear, Detroit. 6. Treatment of posterior presentation of the vertex, E. P. Bernardy, Philadelphia. 7. Relation of local visceral disorders to the delusions and hallucinations of the insane, W. P. Manton, Detroit. 8. Differential diagnosis of hemorrhage, shock and sepsis, Eugene Boise, Grand Rapids. 9. Movable kidney: local and remote results, A. H. Cordier, Kansas City. 10. Pathology and indications for active surgical treatment in contusions of the abdomen, W. G. Macdonald, Albany. 11. Some causes of insanity in women, George H. Rohé, Sykesville. 12. Subject to be announced, John Milton Duff, Pittsburg. 13. Shall hysterectomy be performed in inflammatory diseases of the appendages? L. H. Dunning, Indianapolis. 14. Subject to be announced, Rufus B. Hall, Cincinnati. 15. Subject to be announced, Geo. Ben Johnston, Richmond. 16. Dynamic ileus: with report of cases, J. W. Long, Richmond. 17. Faradic treatment of uterine inertia and subinvolution, Charles Stover, Amsterdam. 18. A plea for absorbable ligatures, H. E. Hayd, Buffalo. 19. Treatment of the stump, J. F. Baldwin, Columbus. 20. Limitations in the teaching of obstetrics and gynecology as determined by state medical examining boards, William Warren Potter, Buffalo. 21. Subject to be announced, Walter B. Chase, Brooklyn. 22. (*a*) The philosophy of drainage; (*b*) Treatment of the pedicle in hysterectomy or hystero-myomectomy in the abdominal method, Geo. F. Hulbert, St. Louis. 23. Removal of the uterine appendages for epilepsy and insanity; a plea for its more general adoption, D. Tod Gilliam, Columbus. 24. Albuminuria of pregnancy, A. Fr. Eklund, Stockholm. 25. Subject to be announced, Lawson Tait, Birmingham. 26. Unnecessary and unnatural fixation of the uterus and its results, James F. W. Ross, Toronto. 27. Sarcoma of the Urethra, Charles A. L. Reed, Cincinnati. 28. Appendicitis as a complication in suppurative inflammation of the uterine appendages, L. S. McMurtry, Louisville. 29. Gunshot wounds of the abdomen with the new gun, J. D. Griffith, Kansas City. 30. Subject to be announced, Walter B. Dorsett, St. Louis. 31. Subject to be announced, W. E. B. Davis, Birmingham. 32. Subject to be announced, E. Arnold Praeger, Los Angeles. 33. Tubo-ovarian cysts with interesting cases, A. Goldspohn, Chicago. 34. Obstruction of the bowels following abdominal section, Geo. S. Peck, Youngstown. 35. Memorial of Dr. Hiram Corson, Traill Green, Easton.

Correspondence is pending concerning additional papers. All titles must be offered before August 25th, when the permanent program goes to press. The executive council directs attention to the following by-law.

PAPERS.

VI. The titles of all papers to be read at any annual meeting shall be furnished to the secretary *not later* than one month before the first day of the meeting.

No paper shall be read before the association that has already been published or that has been read before any other body.

Not more than thirty minutes shall be occupied in reading any paper before the association.

Abstracts of all papers read should be furnished to the secretary at the meeting.

All papers read before the Association shall become its sole property if accepted for publication; and the Executive Council may decline to publish any paper *not handed to the secretary complete* before the final adjournment of the annual meeting.

Dr. Geo. Ben Johnston, 407 E. Grace street, Richmond, Va., is chairman of the committee of arrangements, who should be addressed in regard to hotel accommodations and railway fares.

JOSEPH PRICE, *President.*

William Warren Potter, *Secretary.*

---

The twenty-fourth annual meeting of the American Public Health Association will be held at Buffalo, N. Y., September 15–18, 1896. The Executive Committee has selected the following topics for consideration: 1. The pollution of water supplies. 2. The disposal of garbage and refuse. 3. Animal diseases and animal food. 4. The nomenclature of diseases and forms of statistics. 5. Protective inoculations in infectious diseases. 6. National health legislation. 7. The cause and prevention of diphtheria. 8. Causes and prevention of infant mortality. 9. Car sanitation. 10. The prevention of the spread of yellow fever. 11. Steamship and steamboat sanitation. 12. The transportation and disposal of the dead. 13. The use of alcoholic drinks from a sanitary standpoint. 14. The centennial of vaccination. 15. The relation of forestry to public health. 16. Transportation of diseased tissues by mail. 17. River conservancy boards of supervision. Upon all the above subjects special committees have been appointed. Papers will be received upon other sanitary and hygienic subjects also.

At the date of this announcement the indications are that the next annual meeting will be one of the largest and most successful in the history of the Association. The local Committee of Arrangements has already begun active preparations for the meeting, and it will be materially aided and assisted by the State Board of Health of New York.

An announcement will be made in ample time before the meeting giving full particulars regarding reduced fares on railroads, hotel rates and accommodations, special entertainments to be arranged by the local committee, and the like.

All communications relating to local matters should be addressed to Dr. Ernest Wende, chairman Local Committee of Arrangements, Buffalo, N. Y.

---

The American Dermatological Association will hold its next annual meeting at the Hot Springs of Virginia, September 8, 9 and 10, 1896. Everything will be done to make the meeting a success, and several papers on interesting subjects have been already promised. Dr. White will open a general discussion on the subject, What effect do diet and alcohol have upon the causation and course of the eczematous affections and psoriasis? Dr. Charles W. Allen, of New York, is the secretary.

